# Salt-tolerant and -sensitive alfalfa (*Medicago sativa*) cultivars have large variations in defense responses to the lepidopteran insect *Spodoptera litura* under normal and salt stress condition

**DOI:** 10.1371/journal.pone.0181589

**Published:** 2017-07-18

**Authors:** Yunting Lei, Qing Liu, Christian Hettenhausen, Guoyan Cao, Qing Tan, Weiye Zhao, Honghui Lin, Jianqiang Wu

**Affiliations:** 1 Ministry of Education Key Laboratory for Bio-Resource and Eco-Environment, College of Life Science, State Key Laboratory of Hydraulics and Mountain River Engineering, Sichuan University, Chengdu, China; 2 Key Laboratory of Mountain Ecological Restoration and Bioresource Utilization & Ecological Restoration and Biodiversity Conservation Key Laboratory of Sichuan Province, Chengdu Institute of Biology, Chinese Academy of Sciences, Chengdu, China; 3 Department of Economic Plants and Biotechnology, Yunnan Key Laboratory for Wild Plant Resources, Kunming institute of Botany, Chinese Academy of Sciences, Kunming, China; Louisiana State University College of Agriculture, UNITED STATES

## Abstract

In nature, plants are often exposed to multiple stress factors at the same time. Yet, little is known about how plants modulate their physiology to counteract simultaneous abiotic and biotic stresses, such as soil salinity and insect herbivory. In this study, insect performance bioassays, phytohormone measurements, quantification of transcripts, and protein determination were employed to study the phenotypic variations of two alfalfa (*Medicago sativa*) cultivars in response to insect *Spodoptera litura* feeding under normal and salt stress condition. When being cultivated in normal soil, the salt-tolerant alfalfa cultivar Zhongmu-1 exhibited lower insect resistance than did the salt-sensitive cultivar Xinjiang Daye. Under salinity stress, the defense responses of Xinjiang Daye were repressed, whereas Zhongmu-1 did not show changes in resistance levels. It is likely that salinity influenced the resistance of Xinjiang Daye through suppressing the accumulation of jasmonic acid-isoleucine (JA-Ile), which is the bioactive hormone inducing herbivore defense responses, leading to attenuated trypsin proteinase inhibitor (TPI) activity. Furthermore, exogenous ABA supplementation suppressed the insect herbivory-induced JA/JA-Ile accumulation and levels of *JAR1* (*jasmonate resistant 1*) and TPI, and further decreased the resistance of Xinjiang Daye, whereas Zhongmu-1 showed very little response to the increased ABA level. We propose a mechanism, in which high levels of abscisic acid induced by salt treatment may affect the expression levels of *JAR1* and consequently decrease JA-Ile accumulation and thus partly suppress the defense of Xinjiang Daye against insects under salt stress. This study provides new insight into the mechanism by which alfalfa responds to concurrent abiotic and biotic stresses.

## Introduction

Plants are constantly exposed to various abiotic and biotic stresses in nature [1], including insect herbivory and salt stress. Accordingly, plants have evolved sophisticated mechanisms to adapt to these stresses [2, 3]. Feeding of chewing insects can be perceived by plants, either through perception of certain elicitors in the insect oral secretions (OS) or sensing wound signals [4]. Consequently, insect attack results in changes in plant morphologies and/or chemical compositions, which are mediated by molecular processes such as phytohormone signaling and transcriptomic rearrangements [3, 5]. Among these responses, jasmonic acid (JA) signaling plays a critical role in plant defense against insects. Insect feeding rapidly activates JA biosynthesis, and JA is further converted to JA-isoleucine conjugate (JA-Ile) by the JAR1 enzyme. Binding of JA-Ile to the receptor COI1 activates JA signaling, leading to transcriptional accumulation of defense-related genes, such as proteinase inhibitors, and thus increasing the concentrations of defensive metabolites [6]. Meanwhile, abiotic stress induces a largely different set of plant responses. Excessive soil salinity results in retarded growth and development and highly reduced seed production [7, 8]. Abscisic acid (ABA) signaling is one of the most important regulators in plant tolerance to abiotic stress, such as drought and salinity [9, 10]. Plants impaired in ABA biosynthesis showed increased susceptibility to salt stress at the vegetative stage [11].

Unlike the controlled conditions in laboratories, different stresses may co-occur in nature. Yet, very little is known about how plants adjust their physiology to adapt to concurrent multiple stresses [1, 12–14]. As one of the most common abiotic factors, soil salinity has a strong impact on plant survival and limits agricultural productivity worldwide. Moreover, a growing body of evidence has indicated that salt stress has an impact on plant resistance to insects. For example, salt stress caused accumulation of proteinase inhibitors, which are potent anti-insect proteins, in tomato plants (*Solanum lycopersicum*) [15]. High salinity significantly decreased aphid (*Acyrthosiphon gossypii*) fecundity on cotton plants (*Gossypium hirsutum*) and this was suggested to be due to increased levels of flavonoids [16]. Furthermore, salt stress reduced the resistance of *Iris hexagona* to leaf miners (*Cerodontha iridiphora*) [17] and salt-treated maize plants (*Zea mays*) exhibited decreased levels of herbivory-induced 1,4-benzoxazin-3-one aglycones, a direct defensive metabolite, and volatile compounds, which function as indirect defenses [18]. ABA integrates and fine-tunes both abiotic and biotic stress response signaling networks. For instance, ABA and JA interact to regulate *Solanum dulcamara* responses to drought and insect *Spodoptera exigua* [19]. Additionally, ABA is also a regulator in plant tolerance to herbivore attack. For example, ABA deficient tomato (*Solanum lycopersicum*) showed reduced resistance to the larvae of *Spodoptera exigua* [20] and *Spodoptera littoralis* performed better on ABA-deficient mutant *aba2-1* in Arabidopsis [21].

Alfalfa (*Medicago sativa*) is a perennial forage legume species with great agronomical importance, due to its low production cost, high nutritional value and quality, perennial growth, and nitrogen fixing capability [22]. Compared with many other crops, alfalfa is relatively tolerant to salt stress [7]. Selection of salinity-tolerant germplasm resources, identification of genes involved in regulating plant responses to salt stress, transcriptomic and proteomic analyses, and genome-wide association studies have been carried out in alfalfa [23–27]. However, little is known about the response of alfalfa to insect herbivores and the diversity of the defense responses among different cultivars [28]. In this study, two cultivars with high and low levels of insect resistance were identified among seven cultivars of alfalfa. We found that the insect-susceptible cultivar was previously described as salt-tolerant [26], whereas the insect-resistant one is salt-sensitive [23]. Bioassays, analyses of phytohormones, quantification of gene transcript levels, and determination of trypsin proteinase inhibitor activity were carried out to study the phenotypic variations of these alfalfa cultivars in response to insect *Spodoptera litura* feeding under normal and salt stress conditions. This study provides new insights into the mechanisms by which alfalfa responds to concurrent abiotic and biotic stresses.

## Materials and methods

### Plant growth and sample treatments

Alfalfa (*Medicago sativa*) seeds of seven cultivars (cvs. Zhongmu-1, Longdong, Hexi, Sandli, Eureka, Tianshui, and Xingjiang Daye) were germinated on wet sterile filter paper in Petri dishes, and the seedlings were transferred to 1-L pots 5 days after germination. The plants were cultivated in a greenhouse with a 20 to 28°C temperature range and an 8-h-dark/16-h-light cycle. Four-week-old plants were used for all experiments.

Since insect feeding behavior is very hard to control, simulated herbivory was used for inducing the plants. OS (oral secretions) were collected from around 50 larvae of *Spodoptera litura* (third to fifth instar) reared on alfalfa, mixed and aliquoted to small amounts, and stored at -80°C until use. All simulated herbivory treatments were performed on the third to fifth leaves from the top. Leaves were wounded with a pattern wheel, and 20 μL of *S*. *litura* OS were applied to the wounds (hereafter named W+OS). In the control group, plants were not treated W+OS. For abiotic stress treatment, plants were treated with water or salt: Four-week-old plants were watered with 500 mL of water (named water treatment) or 250 mM NaCl (named salt treatment), thereafter they were watered normally, and one week after water or salt treatment, plants were left untreated or treated with W+OS. For ABA treatment, four-week-old plants were sprayed every day with 0.5% ethanol (v/v) or 2 mM ABA (in 0.5% ethanol), and after one week, W+OS treatments were performed and untreated plants served as comparisons. At specific time points, leaves were excised, immediately frozen in liquid nitrogen and stored at -80°C until use.

### *Spodoptera litura* bioassays

To compare herbivore growth on alfalfa cultivars, freshly hatched *S*. *litura* larvae were initially placed on leaves (15 larvae/plant) of 10 replicated plants for each cultivar, and insects were let feed freely on all plants (plants of each cultivar were placed closely in a lidless container and insects could move from plants to plants); the larval masses were measured at specific times under control and salt-treatment condition. For two-way choice assays, freshly excised leaves of Zhongmu-1 and Xinjiang Daye were scanned on a scanner to determine the leaf areas with ImageJ software (http://rsbweb.nih.gov/ij/), and then halves of Petri dishes were loaded with the leaves of one cultivar, and leaves of the other cultivar with the same areas were placed on the other halves of the Petri dishes. Five *S*. *litura* larvae (3rd instar) were placed into the middle of each Petri dish (n = 5), and after 4 h of feeding, the insect-consumed areas were determined. The leaf consumption ratio was calculated as the insect-consumed areas divided by the initial total leaf areas.

### Analysis of JA, JA-Ile, and ABA concentrations

Phytohormone determination was done according to a method described by Wu et al. [29]. Sample measurements were carried out on a LCMS-8040 (Shimadzu, Japan) equipped with a Shim-pack XR-ODS column (2.0×75 mm, 2.2 μm) (Shimadzu, Japan).

### RNA extraction and quantitative real-time PCR

Total RNA extraction and cDNA synthesis were performed using TRIzol reagent (Invitrogen, USA) and cDNA Synthesis kit (Invitrogen, USA), respectively. For quantitative real-time PCR (qPCR) analysis, five replicated biological samples were used. The analysis was performed on a CFX Connect™ Real-Time PCR Detection System (Bio-Rad, USA) using qPCR Core kits (Bio-Rad, USA). Transcript levels were extrapolated from standard curves that were constructed using the Ct values vs. log (designated concentrations of two-fold dilution series of cDNAs containing seven data points), and individual gene expression values were expressed relative to the expression of the reference gene *EF-1α* [30]. The qPCR primers in this study, which were designed from their homologs in *Medicago truncatula* [31] and tested for their suitability for qPCR, are listed in S1 Table.

### Analyses of trypsin proteinase inhibitor activity and total protein content

Trypsin proteinase inhibitor activity and total protein content were analyzed with a radial diffusion assay and the Bradford assay, respectively, as described by Van Dam et al. [32].

### Statistical analysis

Three-way ANOVAs were used to compare the different treatments for ABA, JA, and JA-Ile, JA pathway-related gene expression and protein content and activity. Two-way ANOVAs and One-way ANOVA were used to analyze bioassays of insect feeding on two alfalfa cultivars with different stresses and seven alfalfa cultivars, respectively. Student *t-*test was used to compare two cultivars with same treatment, including insect leaf consumption ratio and JA/JA-Ile contents with same time point. Tukey HSD tests were used to separate means when ANOVAs were significant. All data are shown as mean ± standard error.

## Results

### Performance of *S*. *litura* larvae on alfalfa varieties

**Fig 1:**
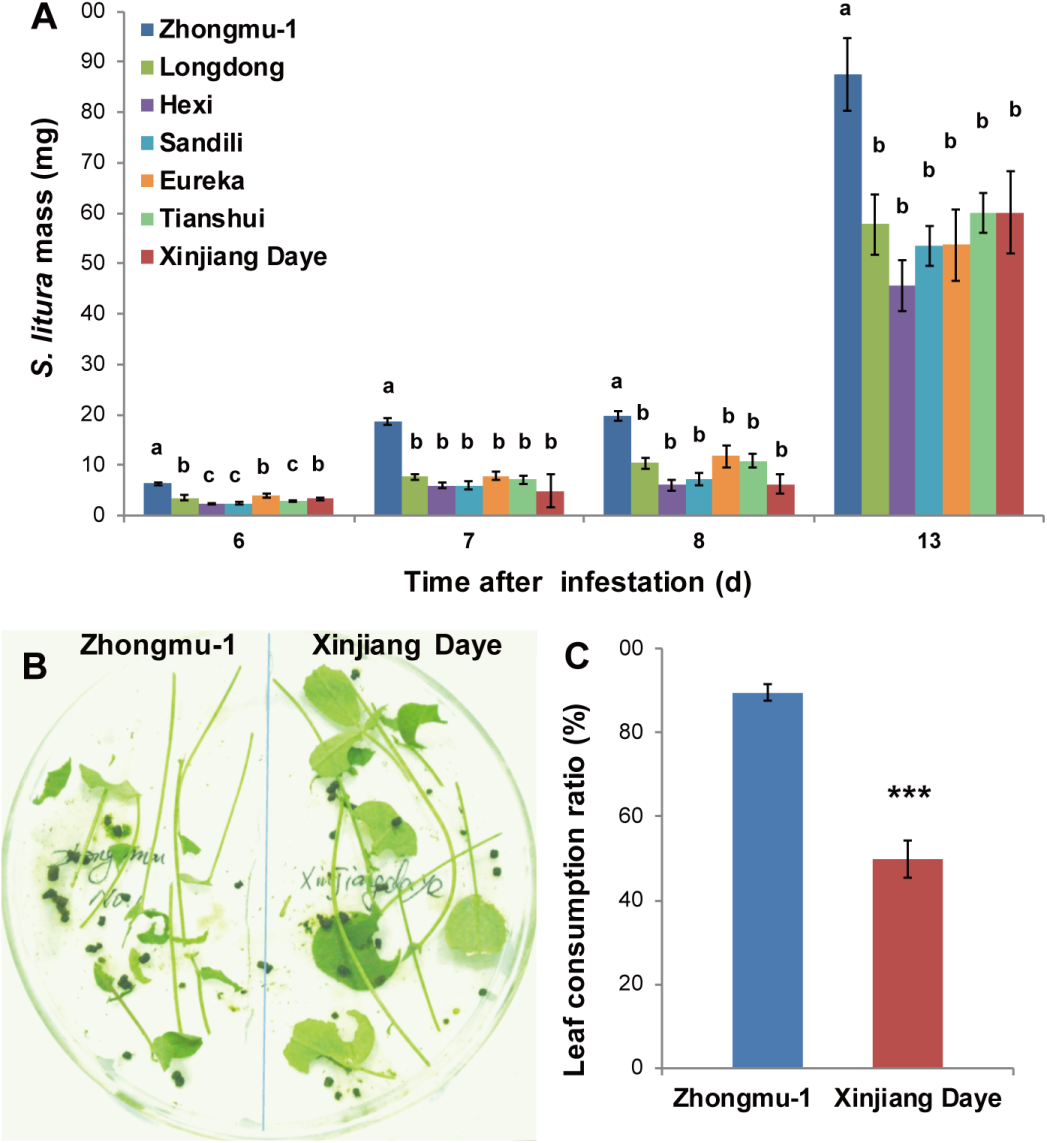
Insect performance on seven different alfalfa cultivars and a two-way choice assay on Zhongmu-1 and Xinjiang Daye. (A) Larval masses of *S*. *litura* feeding on seven different cultivars of alfalfa (n = 150). (B) A photograph and (C) a bar-graph showing the two-way choice test assessing the leaf tissue consumption of *S*. *litura* larvae on Zhongmu-1 and Xinjiang Daye. Different lowercase letters indicate significant differences among different cultivars (Tukey HSD test; P < 0.05); asterisks indicate significantly different levels between two cultivars (paired *t*-test; ***, P < 0.001).

To evaluate the resistance of different alfalfa varieties to the generalist lepidopteran insect *Spodoptera litura*, a no-choice bioassay was performed on seven alfalfa cultivars. We found that *S*. *litura* larvae on Zhongmu-1 grew larger than on the other six cultivars (Fig 1A); for instance, on day 13, the *S*. *litura* larvae on Zhongmu-1 were at least 40% heavier than those on other cultivars (Fig 1A). Notably, compared with Zhongmu-1, the other six alfalfa cultivars were relatively salt-sensitive by comparing the survival rates of seven cultivars of alfalfa with 250 mM NaCl treatment for two months (data no shown). Zhongmu-1 is a salt-tolerant alfalfa cultivar widely grown in China, while Xinjiang Daye was identified as relatively salt-sensitive [23]. To determine whether Zhongmu-1 is preferred over Xinjiang Daye by *S*. *litura*, a two-way choice test was performed, in which the larvae could freely feed on both cultivars. *S*. *litura* showed a strong preference for Zhongmu-1 over Xinjiang Daye (Fig 1B and 1C); after 4 h feeding, almost 90% of leaves from Zhongmu-1 were eaten, whereas only 50% leaf area was consumed from Xinjiang Daye. These results demonstrate that Xinjiang Daye is relatively insect-resistant compared with Zhongmu-1. Thus, Zhongmu-1 and Xinjiang Daye were selected for further experiments to compare their responses to insects under normal and salt stress condition.

### Simulated insect feeding-induced JA and JA-Ile

**Fig 2:**
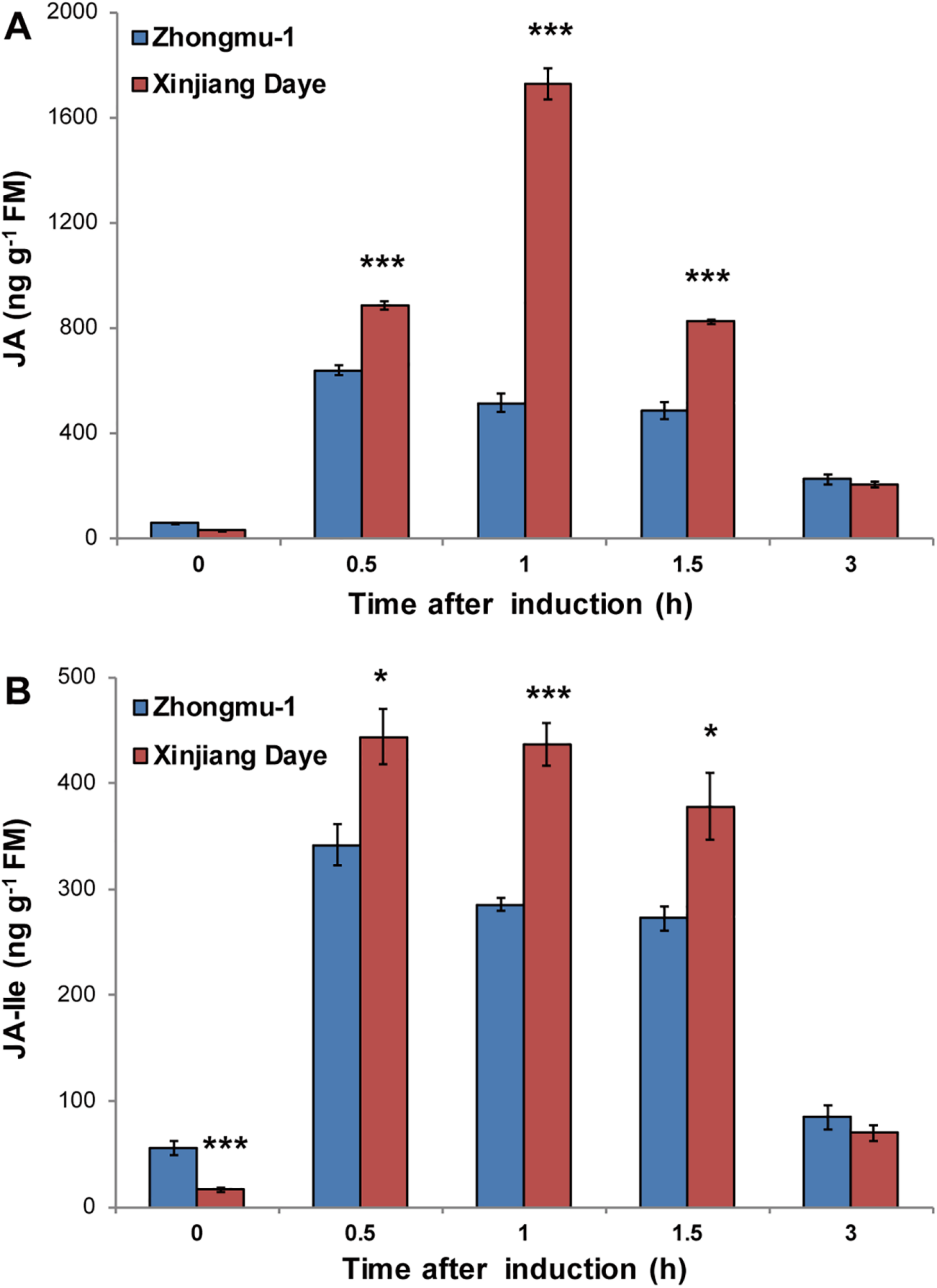
JA and JA-Ile contents in two alfalfa cultivars in response to *S*. *litura* feeding. Zhongmu-1 and Xinjiang Daye were treated with W+OS, and samples were harvested at 0, 0.5, 1, 1.5 and 3 h, and the JA (A) and JA-Ile (B) levels were determined (n = 5). Asterisks indicate significances between two cultivars with the same treatment and time point (Unpaired *t*-test; *, P < 0.05; ***, P < 0.001).

Given the central role of the JA pathway in plant resistance to herbivores, we determined the JA and JA-Ile levels in Xinjiang Daye and Zhongmu-1. In response to simulated *S*. *litura* feeding (W+OS), 38%, 136%, and 69% increased levels of JA were detected in Xinjiang Daye than in Zhongmu-1 at 0.5, 1, and 1.5 h (Fig 2A), respectively. As expected, the patterns of W+OS-induced JA-Ile in these two cultivars were similar to those of JA (Fig 2B). Therefore, consistent with their increased resistance to *S*. *litura*, Xinjiang Daye activates higher levels of JA and JA-Ile after simulated *S*. *litura* feeding than does the insect-susceptible cultivar Zhongmu-1.

### Resistance to *S*. *litura* under normal and salinity condition

**Fig 3:**
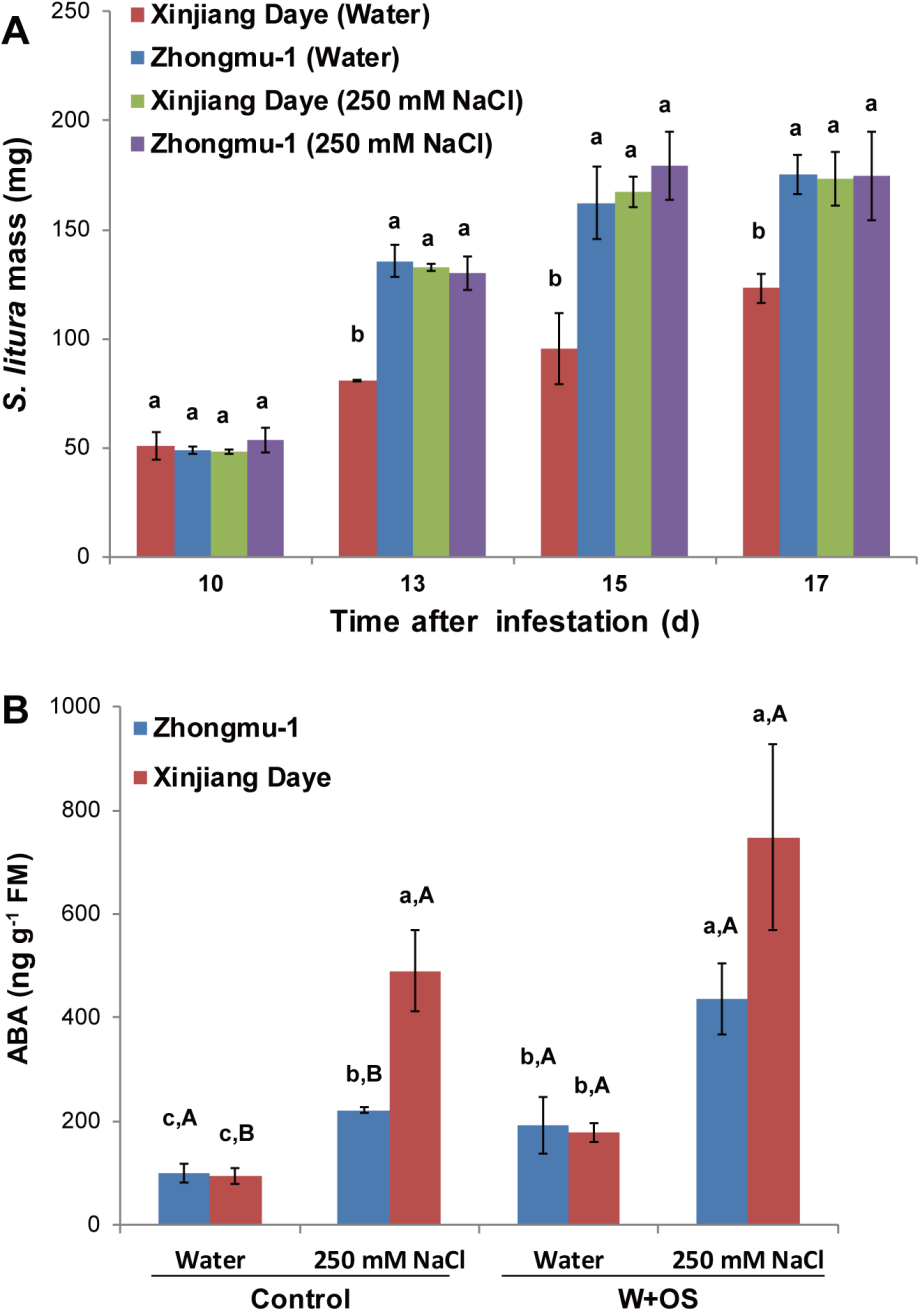
*S*. *litura* growth and ABA contents under normal and salt-stress condition. Zhongmu-1 and Xinjiang Daye were irrigated with 250 mM NaCl or water and thereafter cultivated for a week. (A) Masses of *S*. *litura* feeding on these plants (n = 150). (B) ABA levels in Zhongmu-1 and Xinjiang Daye, 1 h after W+OS treatment (untreated plants served as controls; n = 5). Different lowercase letters represent significant differences among the combinations of abiotic stresses and cultivars. Different uppercase letters indicate significant differences between biotic stresses within the same cultivar and abiotic treatment (Tukey HSD test; P < 0.05).

Previously, Zhongmu-1 and Xinjiang Daye were identified to be relatively salt-tolerant and -sensitive, respectively [23, 26], and our bioassays indicated that they are relatively *S*. *litura*-susceptible and -resistant. Thus, we sought to assess the effect of salinity stress on insect resistance in these alfalfa cultivars, in order to gain insight into the interactions between salt stress- and insect herbivory-induced responses. Zhongmu-1 and Xinjiang Daye were watered with 250 mM NaCl or water, and a week later, *S*. *litura* were infested on these plants. At the 13, 15, and 17 days after infestation, the insects feeding on Xinjiang Daye were bigger when plants were under salt stress compared to normal soil condition, whereas the growth of the insects feeding on Zhongmu-1 were not altered by salt treatment (Fig 3A). Thus, salt stress suppressed insect resistance in the salt-sensitive Xinjiang Daye but did not affect the resistance of the salt-tolerant Zhongmu-1.

Given the importance of ABA signaling in plant adaptation to salt stress, the levels of ABA in these plants were quantified. When being cultivated in normal soil, ABA levels were not very different between Zhongmu-1 and Xinjiang Daye before and 1 h after W+OS treatment, and W+OS did not have an obvious impact on ABA levels in both cultivars (Fig 3B). However, when the plants were cultivated in soil containing excessive salt, compared to their respective controls (95 ng/g and 101 ng/g, respectively), ABA contents increased by 418% in Xinjiang Daye (490 ng/g) and 120% in Zhongmu-1 (221 ng/g). Under the salt-stress condition, W+OS further increased ABA levels in Xinjiang Daye and Zhongmu-1 to 748 and 437 ng/g, respectively (Fig 3B). These data suggest that ABA is likely not important in alfalfa defense against *S*. *litura* under normal soil condition, but may be involved in plant defense against insects under salinity stress, especially in the salt-sensitive Xinjiang Daye.

### Defensive responses to salinity stress in both cultivars

**Fig 4:**
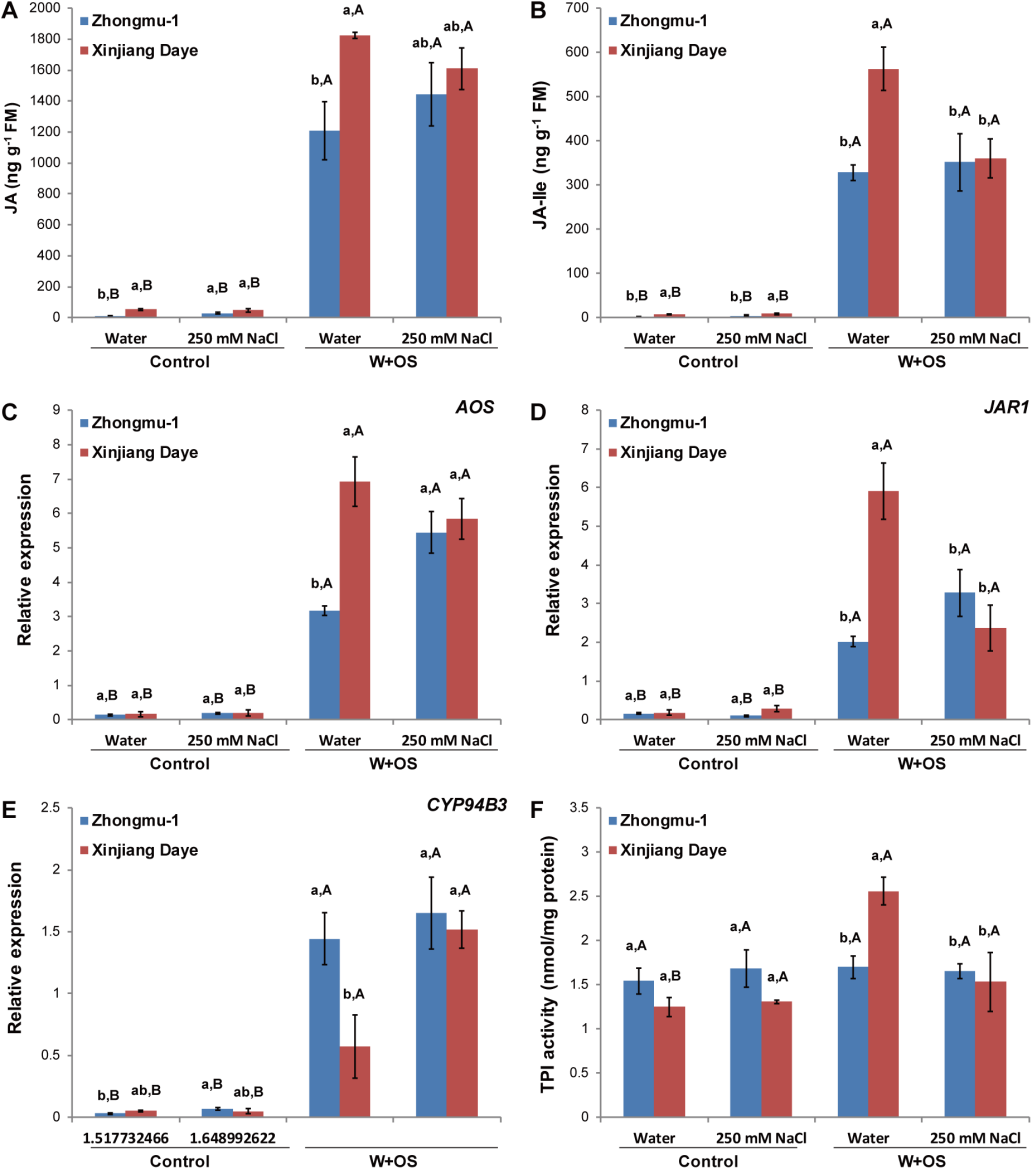
The levels of JA, JA-Ile, *AOS*, *JAR1*, and *CYP94B3*, and TPI activity under normal and salt-stress condition. Zhongmu-1 and Xinjiang Daye were irrigated with 250 mM NaCl or water and thereafter cultivated for a week. Contents of JA (A) and JA-Ile (B) and the relative transcript levels of *AOS* (C), *JAR1* (D), and *CYP94B3* (E) (1 h after W+OS treatment), and TPI activity (48 h after W+OS treatment) (F) in Zhongmu-1 and Xinjiang Daye 1 h after W+OS treatment (untreated plants served as controls; n = 5). Different lower lowercase represent significant differences among the combinations of abiotic stresses and cultivars. Different uppercase letters indicate significant differences between biotic stresses within the same cultivar and abiotic treatment (Tukey HSD test; P < 0.05).

To gain insight into the mechanisms underlying the effect of salt stress on plant resistance to insects, we treated both alfalfa cultivars with simulated *S*. *litura* feeding (W+OS) and analyzed the JA and JA-Ile levels in samples collected 1 h after treatment. Without W+OS treatment, low levels of JA and JA-Ile were detected in water- or salt-treated Zhongmu-1 and Xinjiang Daye (Fig 4A and 4B). One hour after W+OS treatment, both cultivars growing in normal soil exhibited highly elevated JA, and the JA levels in Xinjiang Daye were about 50% higher than those in Zhongmu-1 (Fig 4A). However, in the salt-treated group, JA levels were not significantly different between these two cultivars, since JA tended to decrease in Xinjiang Daye and slightly increased in Zhongmu-1 (Fig 4A). We found that the transcript abundance of a JA biosynthesis gene, *AOS*, exhibited a similar pattern as the JA levels in both cultivars under normal and salt stress condition (Fig 4C). This implies that transcriptional regulation of JA biosynthesis genes may account for the specific JA accumulation in different cultivars. Furthermore, the contents of JA-Ile in these plants displayed a more distinct pattern: salt treatment did not have any effect on W+OS-induced JA-Ile levels in Zhongmu-1, but strongly suppressed W+OS-induced JA-Ile in Xinjiang Daye (36% decreased) (Fig 4B). Thus, salt treatment reduced the resistance of Xinjiang Daye to *S*. *litura* most likely by negatively affecting insect feeding-induced JA-Ile levels; in contrast, salt stress did not have an obvious effect on the levels of JA-Ile in the salt-tolerant cultivar Zhongmu-1 and consequently did not affect its insect resistance.

To further understand how salt treatment suppressed W+OS-induced JA-Ile in Xinjiang Daye, but not in Zhongmu-1, the levels of *JAR1* (*jasmonate resistant 1*), which encodes the key enzyme in JA-Ile biosynthesis [6], were determined in both alfalfa cultivars. Under normal soil condition, W+OS-induced *JAR1* transcript levels in Xinjiang Daye were 261% greater than those in Zhongmu-1; salt treatment significantly suppressed the W+OS-induced accumulation of *JAR1* transcripts in Xinjiang Daye but did not affect the W+OS-induced *JAR1* levels in Zhongmu-1 (Fig 4D). To test whether the degradation of JA-Ile was influenced by salt treatment, the expression of *CYP94B3*, a key enzyme in the oxidative catabolism of jasmonates that catalyzes the formation of 12-OH-JA-Ile from JA-Ile [33], was determined. Under normal soil conditions, after W+OS treatment, *CYP94B3* expression levels in Zhongmu-1 were 1.5-fold higher than in Xinjiang Daye; salt treatment did not have any effect on W+OS-induced *CYP94B3* levels in Zhongmu-1, but strongly elevated W+OS-induced *CYP94B3* expression level (1.6-fold) in Xinjiang Daye (Fig 4E). Therefore, under normal condition, after being attacked by *S*. *litura* insects, the higher JA-Ile levels in Xinjiang Daye likely resulted from the higher *JAR1* and lower *CYP94B3* expression/activity; under salt stress condition, the suppression of JA-Ile levels in Xinjiang Daye was probably because of its strongly decreased *JAR1* expression and highly elevated *CYP94B3* expression/activity. In contrast, in Zhongmu-1, whose JA-Ile contents were not different between normal and salt stress condition, the transcriptional regulation of herbivory-induced *JAR1* and *CYP94B3* was consistently not influenced by salt treatment.

Proteinase inhibitors (PIs), such as trypsin proteinase inhibitors (TPIs), exist in many plant families and are important anti-insect proteins. Binding of PIs with insect midgut proteinases debilitates insect digestion of ingested proteins from plants [34]. In *M*. *truncatula*, TPIs are involved in defense against insect herbivory [35] but whether differences between individual alfalfa cultivars occur or if salt treatment affects TPI activity was not known. Thus, the activity of TPIs after simulated *S*. *litura* feeding was determined in Zhongmu-1 and Xinjiang Daye cultivated in normal soil and soil supplemented with salt (Fig 4F). Consistent with the higher levels of W+OS-induced JA/JA-Ile in Xinjiang Daye, under normal condition, W+OS elicitation resulted in 51% higher TPI activity in these plants compared to Zhongmu-1; in contrast, in the salt-treated group, W+OS-induced TPI activity was diminished in Xinjiang Daye to a level that was similar to that in Zhongmu-1 (Fig 4F).

Insect larval growth is also influenced by plant nutrient content, especially that of total proteins. Thus, we analyzed the total protein levels in both alfalfas (S1 Fig). Under normal or salinity condition without W+OS treatment, the protein content of Zhongmu-1 was higher than that of Xinjiang Daye, and this might at least partly account for the finding that the insects quickly identified the more preferred food source in the choice assay (Fig 1B and 1C). We did not find a strong effect of salinity stress on the protein levels in either cultivar (S1 Fig). After W+OS, these two cultivars showed similar levels of proteins under normal soil or salt stress condition, although their protein contents under salt stress condition were about half of those under normal soil condition (S1 Fig). Thus, it is likely that the protein levels were not the reason for the lower defense levels of Zhongmu-1 than that of Xinjiang Daye under normal soil condition, but this was due to the lower levels of defensive metabolites induced by the JA/JA-Ile in Zhongmu-1 (Fig 4).

### W+OS-induced responses to exogenously supplied ABA

**Fig 5:**
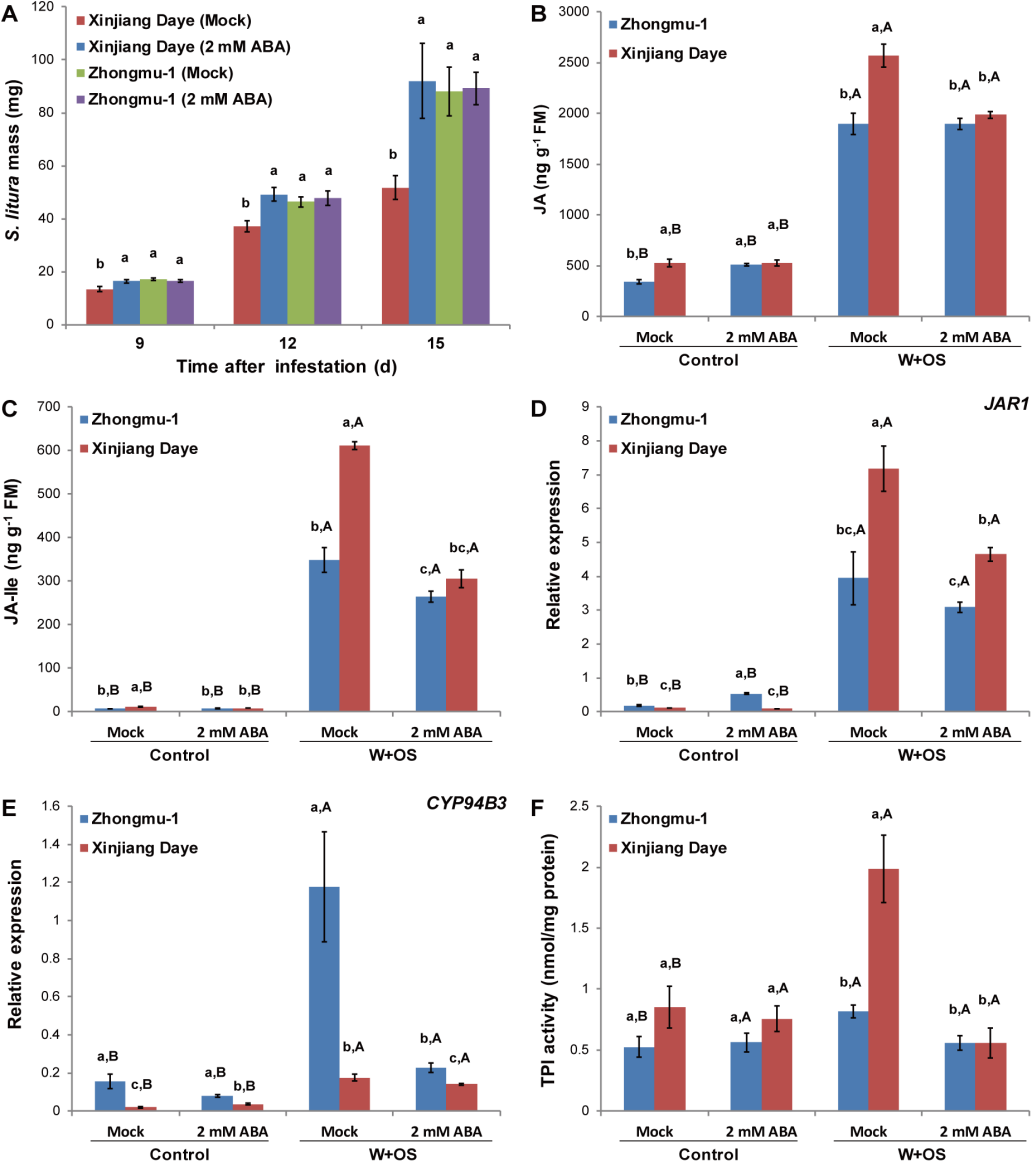
*S*. *litura* growth and the levels of phytohormones JA and JA-Ile, transcripts *JAR1* and *CYP94B3*, and TPI activity after ABA treatment. Zhongmu-1 and Xinjiang Daye were sprayed with 2 mM ABA or 0.5% ethanol (mock) and thereafter cultivated for a week. (A) Masses of *S*. *litura* feeding on these plants (n = 150). Contents of JA (B) and JA-Ile (C) and the relative transcript levels of *JAR1* (D) and *CYP94B3* (E) (1 h after W+OS treatment), and TPI activity (48 h after W+OS treatment) (F) in Zhongmu-1 and Xinjiang Daye (untreated plants served as controls; n = 5). Different lowercase letters represent significant differences among the combinations of abiotic stresses and cultivars. Different uppercase letters indicate significant differences between biotic stresses within the same abiotic treatment and cultivar (Tukey HSD test; P < 0.05).

To test whether salt stress-induced ABA accumulation has an effect on W+OS-elicited JA/JA-Ile levels, which further regulate the levels of defensive metabolites, ABA (2 mM) was sprayed to both alfalfas and plant resistance to *S*. *litura* was determined. Similar to salt treatment, ABA supplementation decreased the resistance of Xinjiang Daye, but had no effect on Zhongmu-1(Fig 5A). Furthermore, supplementation of ABA had almost no effect on W+OS-induced JA/JA-Ile levels in Zhongmu-1, but exhibited a strong suppression effect on Xinjiang Daye (23 and 50% decreased JA and JA-Ile, respectively; Fig 5B and 5C). Consistently, we also found that applying ABA to Xinjiang Daye suppressed W+OS-induced levels of *JAR1* expression and TPI activity, but this effect was not detected in Zhongmu-1, except for *CYP94B3* (Fig 5D and 5F). Furthermore, when both alfalfas were treated with ABA supplementation, no significant changes in W+OS-induced TPI levels were detected in Zhongmu-1, but in Xinjiang Daye, the levels of TPIs were 72% reduced (Fig 5F). These data from ABA supplementation further support the hypothesis that the high levels of ABA suppress JA/JA-Ile and thereby decrease the contents of defensive metabolites in Xinjiang Daye, while ABA has little effect on JA/JA-Ile levels in Zhongmu-1.

## Discussion

Plants rely on different signaling cascades to fine-tune their responses to abiotic and biotic stresses, including soil salinity and insect herbivory. Many studies have focused on plant adaptation to a specific stress, but yet little is known about how plants cope with concurrent multiple stresses. Alfalfa is an important forage crop, but its phenotypic variations in the responses of individual cultivars to salt stress and insect herbivory and the underlying molecular mechanisms are elusive. In this study, we examined the resistance of two alfalfa cultivars under normal and salinity-stress conditions, and we show that the salt-tolerant alfalfa cultivar Zhongmu-1 is susceptible to insect feeding compared with Xinjiang Daye, which is a relatively salt-sensitive cultivar. Furthermore, under salt stress, the resistance of salt-tolerant Zhongmu-1 to insects did not have changes, but Xinjiang Daye had decreased defense levels. These results indicate interactions between salt- and herbivory-induced signaling pathways in alfalfa and show large phenotypic differences among different cultivars.

In plant defense against insects, especially chewing caterpillars, JA signaling plays a critical role [6]. After simulated *S*. *litura* feeding, Xinjiang Daye accumulated substantially greater levels of JA than did Zhongmu-1 (Fig 2A), and consistently, Zhongmu-1 exhibited lower resistance levels than did Xinjiang Daye (Fig 1). Compared with normal condition, under salinity stress, W+OS-induced levels of JA-Ile did not change in Zhongmu-1, but decreased in Xinjiang Daye (Fig 4B). In line with this, *S*. *litura* growth was better on salt-treated Xinjiang Daye than on plants cultivated in normal soil, and Zhongmu-1 did not show changes in resistance to *S*. *litura* (Fig 3A). These data support the notion that JA pathway is the critical factor that determines herbivore defense in plants. Different levels of insect feeding-induced JA/JA-Ile were also found in two accessions of the wild tobacco *Nicotiana attenuata*: Insect *Manduca sexta*-induced JA and JA-Ile levels in the Utah accession were higher than those in the Arizona accession, and because of higher levels of defense-related metabolites, the Utah accession was better defended against *M*. *sexta* [29].

Many metabolic compounds in plants influencing insect resistance are controlled, at least in part, by the JA signaling cascade [5, 34]. Under normal soil condition, in response to W+OS treatment, the activity of TPI was greater in Xinjiang Daye than in Zhongmu-1 (Figs 4F and 5F), and given the high contents of JA/JA-Ile in Xinjiang Daye, the regulation of TPIs is likely to be at least partly modulated by the JA signaling. Although TPIs have not been purified and structurally identified in alfalfa, the expression profiles suggest that TPIs carry anti-insect functions and different cultivars of alfalfa have variations in their contents. Further characterization of TPIs and analysis of more alfalfa varieties may facilitate breeding new insect-resistant cultivars.

ABA is one of the most important regulators in plant adaptation to salt stress, and in response to salt treatment, many plants elevate their ABA levels [9]. Strikingly, after salt treatment, the salt-tolerant cultivar Zhongmu-1 increased by 120% its ABA levels, while the ABA contents in the salt-sensitive Xinjiang Daye were elevated 418% (Fig 3B). Therefore, compared with Xinjiang Daye, ABA signaling likely plays a more important role in the adaptation of Zhongmu-1 to salt stress. The resistance levels of the two alfalfa cultivars were similar under salt-stress condition (Fig 3A), and this was congruent with their W+OS-induced JA-Ile levels (Fig 4B), suggesting that the JA pathway is the predominant regulator of alfalfa defense against insects. It has been shown that ABA has an antagonist effect on the JA pathway through interaction between the ABA receptor PYL6 (RCAR9) with the basic helix-loop-helix transcription factor MYC2, which is the main transcriptional regulator in the JA pathway [14, 36]. In Arabidopsis, ABA-deficient *aba2-1* mutants showed increased resistance to the pathogen *Fusarium oxysporum*, due to upregulated JA-ethylene responsive defense genes [37]. Infection of *Pseudomonas syringae* pv *tomato* DC3000 increased JA levels in Arabidopsis, but this effect was compromised in the ABA-deficient *aba3-1* mutants [38]. In this study, Xinjiang Daye cultivated in high salinity soil exhibited suppressed JA-Ile (Fig 4B) rather than JA contents (Fig 4A) while the salt-resistant Zhongmu-1 did not show this phenotype. Consistently, the JA-Ile biosynthesis gene *JAR1* was repressed and the expression of the JA-Ile degrading enzyme *CYP94B3* was increased by salt treatment in Xinjiang Daye (Fig 4D and 4E). A similar effect was also recently discovered in Arabidopsis: In response to dehydration, elevated ABA suppressed JA-Ile accumulation in WT but not in the ABA-deficient mutant *nced3-2* and this was most likely because of reduced expression of *JAR1* and an increase of the JA-Ile degrading enzyme *CYP94B3* [39]. Our experiment using external application of ABA confirmed that ABA also partly suppressed the defense against insects by inhibiting *JAR1* expression and reducing JA-Ile accumulation and TPI activity in Xinjiang Daye (Fig 5). Genetic analyses are needed to clarify the interactions between JA and ABA signaling in adaptation to simultaneous salinity and herbivory stress in alfalfa. Moreover, although salt stress and ABA supplementation clearly suppressed the levels of W+OS-induced JA-Ile and TPI activity in Xinjiang Daye, TPI activity levels did not completely follow the pattern of JA-Ile. We speculate that in addition to JA/JA-Ile signaling, there might be other pathways controlling TPI and probably other defensive metabolites, which are independent of ABA and/or JA.

Like other organisms, plants have limited energy and resources to maintain growth and development, as well as defense [40]. Phytohormones are believed to play a critical role in modulating the tradeoffs between growth and defense [10, 41, 42]. In consistence with the growth-defense tradeoff theory, the salt-sensitive cultivar Xinjiang Daye and salt-tolerant Zhongmu-1 are insect-resistant and -susceptible, respectively, and the interaction between ABA and JA pathway are likely to be involved in the balance/tradeoff between salt and insect stress adaption/resistance. In *Solanum dulcamara*, drought stress increased plant resistance to *Spodoptera exigua*, and JA and ABA interaction was also proposed to be involved in optimizing plant response to combined drought and herbivory [19]. The complexity of tradeoffs among growth, abiotic defense, and biotic defense and variation of plants in adapting to multiple stresses can be demonstrated from protein content analysis: The salt-tolerant Zhongmu-1 showed a greater protein content than did the salt-sensitive Xinjiang Daye under normal soil condition, and this pattern persisted even after salt treatment (S1 Fig); however, after W+OS treatment, in normal soil, protein level of Xinjiang Daye increased and that of Zhongmu-1 remained the same, but plants in salinity soil all showed decreased levels of proteins (S1 Fig).

Variations among individuals, populations, ecotypes, etc. are the driving force of evolution and are the basis for adaptation to different environmental conditions. Further studies on these two and other alfalfa cultivars will not only shed light on mechanisms of alfalfa adaptation to salt stress and defense against insects, but also reveal the genetic basis of their contrary tolerance to salt stress and defense against insect herbivores. Marker assisted breeding will also be feasible to create new salt- and insect-resistant alfalfa varieties.

## Supporting information

S1 Fig Protein content in two cultivars under normal and salt stress condition. (DOCX)

S1 Table Primers used for qPCR. (DOCX)
