# Supplementary material for: Salt-tolerant and -sensitive alfalfa (Medicago sativa) cultivars have large variations in defense responses to the lepidopteran insect Spodoptera litura under normal and salt stress condition
Source: PLoS One. 2017 Jul 18;12(7):e0181589. doi: 10.1371/journal.pone.0181589 (PMC5515460; doi:10.1371/journal.pone.0181589)
Supplement: S1 Fig — (DOCX) [file pone.0181589.s001.docx]

**S1 Fig. Protein content in two cultivars under normal and salt stress condition.**

After growing 40 days in soil, Zhongmu-1 and Xinjiang Daye were irrigated with 500 mL of water or 250 mM NaCl, and thereafter cultivated for a week. The protein contents were measured 48 h after W+OS treatment (untreated plants served as controls; n =5). Different lowercase letters represent significant differences among the combinations of abiotic stresses and cultivars. Different uppercase letters indicate significant differences between biotic stresses within the same cultivar and abiotic treatment (Tukey HSD test; P < 0.05).
