# Supplementary material for: Salt-tolerant and -sensitive alfalfa (Medicago sativa) cultivars have large variations in defense responses to the lepidopteran insect Spodoptera litura under normal and salt stress condition
Source: PLoS One. 2017 Jul 18;12(7):e0181589. doi: 10.1371/journal.pone.0181589 (PMC5515460; doi:10.1371/journal.pone.0181589)
Supplement: S1 Table — (DOCX) [file pone.0181589.s002.docx]

**S1 Table. Primers used for qPCR**

| **Name** | **Description** | **Primer sequences ^a^** | **Accession number** |
| --- | --- | --- | --- |
| *EF-1α* | Elongation factor 1-alpha | GCACGCTCTTCTTGCATTTACT | XM_003618727.2 |
|  |  | GGGCTTGTCTGTGGGTCTCTT |  |
| *AOS* | Allene oxide synthase | CTCTTGAGGAAGCAGAGCGT  TATGCAACCTCACTCCACCG | AJ316561.1 |
| *JAR1* | Jasmonate resistance 1 | GGTGTGACAGTAGCAACAGC | XM_013589219.1 |
|  |  | AGGTGGCAGTAGAGTGATTGG |  |
| *CYP94B3* | Jasmonoyl-isoleucine-12-hydroxylase | AACCAGAGCGGTGGTTTGAA  TCTTGGACCGGCCTGAAAAA | XM_003601379.2 |

^a^ Forward (upper line) and reverse (lower line) primer sequences.
